# Cord-Blood Natural Killer Cell-Based Immunotherapy for Cancer

**DOI:** 10.3389/fimmu.2020.584099

**Published:** 2020-10-22

**Authors:** Xiaoyan Zhao, Li Cai, Yu Hu, Huafang Wang

**Affiliations:** Department of Hematology, Union Hospital, Tongji Medical College, Huazhong University of Science and Technology, Wuhan, China

**Keywords:** immunotherapy, natural killer cells, umbilical cord blood, cancer, cytotoxicity

## Abstract

Natural killer (NK) cells are a predominant part of innate immune cells and play a crucial role in anti-cancer immunity. NK cells can kill target cells nonspecifically, and their recognition of target cells is not restricted by the major histocompatibility complex. NK cells also fight against tumor cells independently of antibodies and prior activation. Of note, umbilical cord blood (UCB) is a rich source of NK cells. Immunotherapies based on UCB-derived NK cells are becoming increasingly researched, and the investigations are producing encouraging results. In recent years, non-modified and modified UCB-derived NK cells have been successfully developed to fight against tumor cells. Herein, UCB-derived NK cell-based immunotherapy is a potential strategy for the treatment of cancer in the future. In this review, we focus on discussing the biological characteristics of UCB-derived NK cells and their application prospects in anti-tumor immunotherapy, including the latest preclinical and clinical researches.

## Introduction

Adoptive cellular immunotherapy, including chimeric antigen receptors (CARs)-T cells-based and natural killer (NK) cell-based therapies, has been used to cure cancer, making significant improvements in the prognosis of patients (1–3). NK cells are large granular lymphocytes in morphology and are the core cells of the innate immune system. Because NK cells – which are the first line of defense against cancer cells and virus infection and can kill tumor cells without antigen sensitization and antibody involvement – play an important role in anti-tumor immunity, they have been used in the immunotherapy of malignant tumors in recent years (4, 5). NK cells can identify tumor cells *via* unique mechanisms that depend on a set of stimulatory and inhibitory receptors, such the NKp30, NKp46, NKG2D, and NKG2A, and these receptors, acting as switches, determine whether NK cells are activated to kill target cells. Once activated, NK cells release perforin and granzyme; perforin perforates the surface of target cells, facilitating granzyme B to induce the apoptosis of target cells. Simultaneously, NK cells can also secrete amounts of cytokines, including IFN-γ and TNF-α, which act on the target cells directly or further activate other types of immune cells. Moreover, NK cells enable target cells to be programmed for apoptosis through Fas/FasL or TRAIL (4, 5).

NK cells are derived from multiple platforms, including peripheral blood (PB), Umbilical cord blood (UCB), induced pluripotent stem cells, and embryonic stem cells. The latest report in 2018 revealed that more than 600,000 UCB units from around the world were stored in a rich UCB bank, and this amount is expected to shall continue to rise (6). Unlike PB, UCB can be conveniently collected and frozen, making it readily obtainable (7). Recently, UCB is used as a source of hematopoietic stem cells (8). Of note, UCB has also been regarded as an allogeneic and off-the-shelf source of NK cells (9). A variety of expansion methods have been exploited to elevate the number and activity of NK cells to satisfy clinical use (10, 11). Given that preclinical and clinical results of UCB-derived NK cells-based therapies have been encouraging (12–14), it is reasonable to infer that this form of immunotherapy is attractive and promising. In this review, we discuss the biological characteristics of UCB-derived NK cells and their application prospects in anti-tumor immunotherapy, including in the latest preclinical and clinical researches.

## An Overview of NK Cell Basic Biology

A basic function of NK cells is to eliminate cells expressing down-regulated and missing major histocompatibility complex class I molecules (15), additionally, NK cells are also activated by tumor cells overexpressing NK cell-activating receptors ligands (4, 16). The activation of NK cells occurs *via* intricate interactions among activating, co-stimulatory, and inhibitory receptors (4, 17). NK cell receptors include Non-HLA-specific receptors (activating: NKp30, NKp46, NKp44, NKG2D, CD16; inhibitory: PD-1, Siglec-7, TIGIT, TIM-3, Tactile, IL1R8; Coreceptors: CD59, NTB-A, NKp80, DNAM-1, 2B4) and HLA-specific receptors (activating: KIR2DS1, KIR2DS2/3, KIR2DL4, KIR2DS4, KIR2DS5, KIR3DS1, NKG2C; inhibitory: NKG2A, KIR2DL1, KIR2DL2/3, KIR2DL5, KIR3DL1, KIR3DL2, ILT2/LIR-1, LAG-3) (16, 17). These receptors are very essential to NK cells and can be used as immune checkpoints and therapeutic targets for NK cell-based immunotherapy (18). Some T cell surface receptors, such as PD-1, TIGIT, and TIM-3, are also expressed on NK cells, and are potentially also valuable in mediating the anti-tumor activity of NK cells (19, 20). The studies demonstrated that the number of NK cells and their surface activating receptors (NKp30, NKG2D) were downregulated in multiple malignant tumors, like acute myeloid leukemia (AML) and multiple myeloma (MM), whereas, the inhibitory receptors of NK cells were overexpressed in these tumors (21, 22). Any disorder in the expression of these receptors would render NK cells unable to activate normally, and the NK cells’ ability to secrete cytokines and chemokines, and cytotoxicity would be affected as well. Researchers have, therefore, made attempts to harness “off-the-shelf” NK cells, such as UCB-derived NK cells, to treat patients with cancer (23–25).

## The Characteristics of UCB-Derived NK Cells

### Advantages of UCB-Derived NK Cells

In UCB, NK cells account for about 30% of lymphocytes; by contrast, they account for 10% of lymphocytes in PB counterparts (26, 27). Also, the immunophenotype of UCB-derived NK cells is CD3^-^CD56^+^, which is roughly classified as the less differentiated CD56^bright^ and mature CD56^dim^ NK cells in a broad sense; studies have indicated that the proportion of CD16^-^CD56^bright^ NK in UCB is higher than that in PB (27, 28). UCB is easy to collect and frozen (29). Additionally, NK cells are the first immune cells to recover after hematopoietic stem cell transplantation (HSCT), suggesting they play a critical role in immune reconstruction post-transplantation (30). UCB-derived NK cells are younger and have a stronger proliferation potential than the PB counterparts (28). There are few T cells in UCB, and most of them are immature, which reduces the occurrence of graft versus host disease (GVHD) (31, 32). Moreover, as one of the bone marrow (BM) homing receptors, the expression levels of CXCR4 in UCB-derived NK cells are higher than those in PB-NK cells, which suggests that UCB-derived NK cells have a better bone marrow homing ability (27). According to Nomura A et al., UCB CD56^bright^ NK cells secrete higher levels of IFN-γ and increased CD69 when stimulated with IL-12 and IL-18, compared with PB-NK cells (33).

### Drawbacks of UCB-Derived NK Cells

Unexpanded UCB-derived NK cells have some limitations, including availability in low numbers due to the small volume of the UCB unit and immature function (27). In addition, UCB-derived NK cells’ cytotoxicity against K562 cells is weaker than that of PB-acquired NK cells, although their degranulation function is identical to that of PB-derived NK cells (34). Reportedly, UCB-derived NK cells possessing lower expressions of CD16, KIRs, DNAM-1, NKG2C, IL-2R, and granzyme B and higher expression of NKG2A are less potent in killing target cells, compared to PB-derived NK cells (27, 34). However, when they are stimulated by cytokines, the cytotoxicity of UCB-derived NK cells is comparable to that of PB-acquired NK cells (28, 35). Thus, to overcome these shortcomings, a diverse range of approaches were developed to expand NK cells *in vitro*.

### Expansion of UCB-Derived NK Cells

Researchers are committed to achieving large-scale expansion in their numbers and enhancing the activity of UCB-derived NK cells *in vitro* through a variety of methods. IL-2-alone was previously used to expand NK cell, but this approach was not as successful as anticipated and needed to be further improved. Subsequently, multiple cytokines-only approaches, including IL-2, IL-12, IL-15, and IL-18, were, therefore, used to satisfactorily expand NK cells (11, 36). Besides, artificial antigen-presenting cells combined with cytokines have been utilized to expand NK cells *in vitro*, with satisfactory purity and number of NK cells. For example, Shah N et al. established a method for purifying and expanding a significant amount of NK cells, that is, NK cells were expanded by using K562-based cells expressing membrane-bound IL-21 (10). In another method, 6092 × 10^6^ expanded NK cells were obtained from 1 ml UCB unit in 21 days using irradiated Epstein-Barr virus-transformed lymphoblastoid cell lines and continuous IL-2 stimulation (37). These reported expansion methods need to ensure that K562 and lymphoblastoid cells do not proliferate for security.

## UCB-Derived NK Cells in Preclinical Applications

### Non-Modified UCB-Derived NK Cells

Based on the above methods of expansion, preclinical studies have been performed to verify the activity of expanded UCB-derived NK cells. Investigations have shown that UCB-derived NK cells expanded *in vitro* were cytotoxic to primary breast cancer cells and producing high levels of IFN-γ and TNF-α (7). Also, tests have also shown that UCB CD34^+^-derived NK cells were more cytotoxic to cervical tumor cells, which were not restricted by HLA-ABC expression, and the levels of degranulation of NK cells are higher, comparing to PB-NK cells’ (38).

Additionally, UCB-derived NK cells combined with antibodies yields an increased anti-tumor effect. Behnaz Valipour et al. revealed that UCB-derived CD16^+^ NK cells combined with an anti-CD47 antibody fought against acute lymphoblastic leukemia cells more effectively and produced higher levels of IFN-γ and CD107a than UCB-derived NK cells alone (39). Another study found that UCB-derived NK cells were against colorectal cancer cell (CRC), and bevacizumab could further enhance their ability to kill CRC (40). Besides, a humanized anti-NKG2A immunoglobulin G (IgG) 4-blocking mAb (monalizumab) has been developed, which could enhance the activity of NK cells (41).

### Modified UCB-Derived NK Cells

#### Enhancing Activation

**Figure 1:**
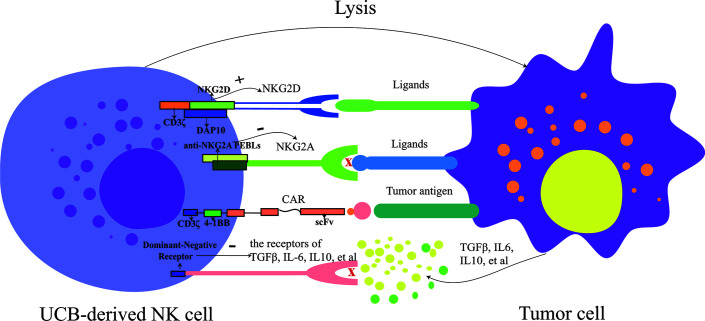
The strategies for modifying UCB-derived NK cells include enhancing activation (such as: increase the expression of activating receptor: NKG2D and decrease the expression of inhibitory receptor: NKG2A), targeting tumor antigen and overcoming the immunosuppressive tumor microenvironment (such as IL-6, IL-10, and TGFβ). PEBLs, anti-NKG2A protein expression blockers; CAR, chimeric antigen receptor; UCB, umbilical cord blood; NK cells, natural killer cells.

Strategies that increase the expression of activating receptors or decrease the inhibitory receptors of NK cells have been explored to enhance the activity of them (42, 43). Upregulating the expression of activating receptors of NK cells could enhance the activity of NK cells, such as NKG2D (**Figure 1**). One study indicated that the CAR-NKG2D-receptor were successfully modified into PB-derived NK cells, and retroviral NKG2D-DAP10-CD3ζ was transduced into NK cells, increasing the expression of NKG2D and enhancing the activity of NK cells. NKG2D delivered signals *via* phosphorylated DAP10, contributing to the expression of downstream signaling molecules, and, therefore, playing a cytotoxic role; this research also demonstrated that the transduced NK cells were more cytotoxic to tumor cells than mock-transduced NK cells and that NKG2D triggered signal transduction and increased cytokine secretion of NK cells (42). It has, likewise, been demonstrated that CAR-PD-1-NKG2D-41BB boosts the capacity of NK92 against lung cancer (44). For UCB-derived NK cells, the expression of NKG2D in expanded NK cells was elevated *in vitro*, and NKG2D mediated the cytotoxicity of them against tumor cells (10, 45, 46). And, the correlation between the expression levels of NKG2D-ligand and susceptibility to NKG2D-DAP10-CD3ζ–bearing NK cells was not significant (42). Hence, we speculate that UCB-derived NK cells can also be genetically modified using CAR (NKG2D-DAP10-CD3ζ), and their cytotoxicity against tumor cells will be enhanced significantly.

Besides, NK cells can also be activated by reducing the expression of inhibitory receptors, such as NKG2A, which can serve as a potential checkpoint for NK cell-based therapy (**Figure 1**). To reduce the expression of NKG2A, anti-NKG2A antibody and anti-NKG2A protein expression blockers (PEBLs) have been used. In patients with leukemia, the cytotoxicity of NK cells has been restored by blocking the expression of NKG2A (47, 48). Takahiro Kamiya and colleagues revealed that HLA-E was overexpressed in multiple tumor samples, and anti-NKG2A PEBLs boosted the cytotoxicity of PB-NK cells against tumor cells expressing HLA-E, compared to the anti-NKG2A antibody Z199. And this investigation also found that NKG2A^null^ NK cells had more potent antitumor properties in a mice model than NKG2A^+^ counterparts (49). Because the expression of NKG2A is relatively higher in UCB-derived NK cells than in PB-NK cells, it would be fascinating to know which of PB or UCB would fare better if antibodies or PEBLs were also used to block the expression of NKG2A. No study has answered this question so far, making further research a necessity. However, it would be reasonable to speculate that combining UCB-derived NK cells with anti-NKG2A molecules or modifying them with CAR-NKG2A would present much more potent anti-tumor activity than using UCB-derived NK cells only. Still, experimental verifications are required to back this theoretical thinking up.

#### Targeting Tumor Antigen

CAR-NK cells produced from the NK92 cell line and primary NK cells are increasingly numerous, showing how CAR-NK cells exert powerful anti-cancer effects (50, 51). Various CARs have been engineered into NK-92 cells to kill specifically CD19, EpCAM, or CD33-positive tumor cells, and these CAR-NK cells have shown high anti-tumor effects (52–54). In recent years, UCB-derived NK cells have been modified *via* CARs (**Figure 1**). Although the number of studies on UCB-derived CAR-NK cells is relatively small so far, existing results are striking and promising. For instance, NK cells derived from the CD34^+^ hematopoietic progenitor stem cells of UCB units could be successfully engineered to express CAR-CD19 using co-culture them with a feeder stroma of murine OP9-DL1 cells in the presence of human recombinant cytokines (55). Another study revealed a novel method that a retroviral vector incorporating the CAR-CD19 gene, IL-15 gene, and inducible caspase-9-based suicide gene (iC9) can be successfully transduced to UCB-derived NK cells (12). In this study, CAR-CD19 was designed to kill CD19 positive cells specifically, IL-15 was used to maintain the survival of NK cells and promote their proliferation, and iC9, as a safety switch, was pharmacologically activated to eliminate transduced cells as needed (56). CAR-transduced NK cells have shown a remarkable ability to kill CD19-positive cells and leukemic cells and significantly prolonged the survival time of Raji xenogeneic mice (12). L Herrera et al. found that UCB-derived CAR-CD19-NK cells generated by transducing CAR-CD19 plasmids to UCB-derived NK cells under IL-2 and IL-15 stimulation were more capable of degranulation when co-cultured with CD19 positive cells, compared to non-modified (57). In the future, except for targeted-CD19 antigen, more types of CARs should be gradually engineered into UCB-derived NK cells.

#### Overcoming the Immunosuppressive Tumor Microenvironment

In addition to focusing on increasing the number of NK cells and enhancing their killing ability, it is also necessary to explore strategies to overcome immunosuppressive tumor microenvironment, such as IL-6, IL-10 and TGFβ, that could mediate immune evasion of tumor cells for adoptive NK cells-based immunotherapy (58). Eric S Yvon et al. revealed that the function of UCB-derived NK cells including the ability to kill glioblastoma tumor cells, the secretion of IFN-γ and perforin, and the expression levels of NKG2D/DNMA1, were maintained in the presence of TGFβ *via* engineering a dominant negative TGFβ receptor II into them (59) (**Figure 1**). Moreover, another study also demonstrated UCB-derived NK cells modified genetically by the novel TGFβ dominant-negative receptor exhibited higher cytotoxic activity against neuroblastoma in a TGFβ-rich environment *in vitro* and superior progression-free survival in mice, compared to unmodified controls. More remarkably, the researchers found that in contrast to RBDNR (containing the truncated TGFβRII domain) and NKCT (containing the truncated TGFβRII domain fused to a synthetic Notch-like receptor coupled to RELA) transduced NK cells, NKA-transduced (containing the truncated TGFβRII domain fused to the DAP12 NK activation motif) UCB-derived NK cells persisted longer in mice and dramatically improved the progression-free survival of mice (60). Nevertheless, UCB-derived NK cells engineered by other types of modified immunosuppressive cytokines receptors, such as modified IL-10 receptor, need to be further developed.

## UCB-Derived NK Cells in Clinical Applications

Due to the promising outcomes of pre-clinical studies, some clinical trials are being performed to evaluate the safety, feasibility, and efficacy of UCB-derived NK cells in the treatment of malignant tumors (**Table 1**). As shown in **Table 1**, different application strategies of UCB-derived NK cells are being explored in phase 1 or 2 clinical trial. Non-modified or modified UCB-derived NK cells could be used as maintenance therapy after chemotherapy, or combined with autologous HSCT or UCB stem cell transplantation as a “bridging transplantation” strategy. The optimal time of UCB-derived NK cells infusion has not been determined in regimen. Alternatively, UCB-derived NK cells could be also infused before or after transplantation (**Table 1**). It is not clear yet whether the sequence of NK cell infusion makes any difference to the prognosis of patients. Outcomes from clinical trials will tell us more when the results become available. Interestingly, so far, the findings of three clinical trials based on UCB-derived NK cells have been published, and the results are encouraging.

**Table 1:** Umbilical cord blood (UCB)-derived natural killer (NK) cells-based therapies were used currently in the clinical trials.

Clinical trial identifier	Disease	Trial phase	The number of estimated Enrollment	Type of transplant	Conditioning	The date of NK infusion	Sponsor
NCT03019640	B-Cell NHL	Phase 2	40	auto-HSCT	Carmustine +etoposide +cytarabine+ melphalan+lenalidomide	Day −5 before transplantation	M.D. Anderson Cancer Center
NCT02280525	Leukemia, Lymphoma	Phase 1	8	non-HSCT	Flu+CY+lenalidomide± rituximab	Day 0	M.D. Anderson Cancer Center
NCT00354172	Leukemia, MDS	Phase 2	16	UCB-SCT	CY+Flu+TBI	Day −5 before transplantation	Masonic Cancer Center, University of Minnesota
NCT02955550	MM	Phase 1	15	auto-HSCT	Melphalan	Days +7 to +14 after transplantation	Celularity Incorporated
NCT02781467	AML	Phase 1	10	non-HSCT	CY+Flu	Day 0	Celularity Incorporated
NCT04347616	AML	Phase 1, Phase 2a	24	non-HSCT	CY+Flu	Day 0	Radboud University
NCT01619761	ALL, AML, CLL. HL, MDS, NHL, SLL	Phase 1	13	UCB-SCT	Flu, melphalan, lenalidomide ± rituximab	Day −2	M.D. Anderson Cancer Center
NCT01729091	MM	Phase 2	72	auto-HSCT	Elotuzumab+lenalidomide+Melphalan	Day −5 before transplantation	M.D. Anderson Cancer Center
ChiCTR2000030622	AML	Phase 1	20	non-HSCT	NR	Two days after consolidation chemotherapy	PLA Air Force General Hospital, China
NCT03056339	CD 19 positive B-lymphoid malignancies (ALL, NHL, CLL)	Phase 1, Phase 2	36	non-HSCT	Flu+CY	Day 0	M.D. Anderson Cancer Center
NCT01729091	MM	Phase1, 2	72	auto-HSCT	melphalan+lenalidomide	Day −5 before transplantation	M.D. Anderson Cancer Center
EudraCT number 2010-018988-41	AML	Phase 1	NR	non-HSCT	Flu+CY	Day 0	Radboud Medical Centre, Nijmegen, Netherlands

AML, Acute myeloid leukemia; NHL, Non-Hodgkin’s Lymphoma; ALL, acute lymphoblastic leukemia; MDS, myelodysplastic syndromes; CLL, chronic lymphocytic leukemia; SLL, small lymphocytic lymphoma; HL, Hodgkin’s Lymphoma; MM, Multiple Myeloma; auto-HSCT, autologous hematopoietic stem cell transplantation; UCB-HSCT, umbilical cord blood-hematopoietic stem cell transplantation; non-HSCT, no combined hematopoietic stem cell transplantation; CY, cyclophosphamide; Flu, fludarabine; TBI, total body irradiation; NR, no report.

### Non-Modified UCB-Derived NK Cells

Non-modified UCB-derived NK cells were used in two clinical trials. One clinical trial indicated that NK cells expanded from UCB CD34^+^ hematopoietic stem cells and progenitor cells achieved successful adoptive transfer in elderly patients with AML, and these patients were in morphologic complete remission post treatment with no GVHD and NK cell infusion-related toxicities, such as cytokine release syndrome (CRS). And in this study, they found chimerism of NK cells up to 21% in PB and up to 3.5% in BM at day 6 to 8. Meanwhile, rapid maturation of UCB-derived NK cells with activating receptors was observed *in vivo*. It was worth mentioning that two of four patients with minimal residual disease (MRD) in BM before NK cells infusion, became MRD negative, which lasted for 6 months, suggesting that UCB-derived NK cells might contribute to the transformation from MRD positive to MRD negative in patients (13).

Besides, UCB-derived NK cells can also be combined with HSCT to treat patients with hematologic malignancies (14). One study indicated that relapsed/refractory MM patients, after the treatment of UCB-derived NK cells (day -5) combined with auto-HSCT (day 0), subsequently achieved very good partial remission with no GVHD and NK cell transfusion-related toxicities. With a median follow-up of 21 months, unfortunately, 4 patients have progressed or relapsed, 2 of whom have died. In the said study, the duration of the UCB-derived NK cells *in vivo* did not exceed 26 days. And UCB-derived NK cells with an activated phenotype (NKp30^+^ and NKG2D^+^) were detected *in vivo* in 6 patients (14), and there was expression of the inhibitory receptor NKG2A on the adoptively transferred UCB-derived NK cells, which suggested a novel combination-therapy strategy with NKG2A blocking antibodies.

### Modified UCB-Derived NK Cells

Up to now, CAR-T cell-based therapy has been used to treat patients with leukemia, MM, and lymphoma, and these patients have benefited from the therapy (61–63). However, the use of CAR-T cell therapy has also resulted in some side effects, including severe CRS, neurotoxicity, and on-target/off-tumor (64, 65). In contrast, NK cells are less likely to lead to these shortcomings. Evidence suggests that CRS is caused by TNF-α, IFN-γ, IL-6, and IL-1 secreted by CAR-T cells. NK cells, on the other hand, secrete IL-3 and the granulocyte-macrophage colony-stimulating factor, which are very unlikely to induce CRS (65, 66). Besides, allogeneic T cells may cause GVHD to pass through native αβTCR, while allogeneic NK cells may not (67). To date, only one clinical investigation based on CAR-UCB-NK cells has published its findings. This phase 1 and 2 trial demonstrated that majority of the patients with CD19-positive Lymphoid tumors who received the CAR-(anti-CD19, CD28.CD3ζ, IL-15, and inducible caspase 9)-UCB-NK cell therapy achieved a rapid response to treatment, and CAR-NK cells maintained their persistence at low levels for at least 12 months. No GVHD, CRS, neurotoxicity, hemophagocytic lympho-histiocytosis and increased the levels of inflammatory factors have been observed in any of the patients thus far. However, a high-grade transient myelotoxicity was observed in patients, possibly due to lymphodepleting chemotherapy (3). Interestingly, CAR-NK cells could be detected in patients who did not have a response or who had a relapse, despite the expression of CD19 in the tumor cells, suggesting that there might be immune escape, such as inducing the exhaustion of CAR-NK cells, but this work has not yet been done. It is worth to further study.

### The Limitations of UCB-Derived NK Cells in Clinical Applications

To sum up, UCB-derived NK cells can induce response, with no serious adverse reactions and GVHD. Whereas, the following problems have been encountered in clinical trials, and further studies should be performed to address these issues. First of all, after the infusion of UCB-derived NK cells, the duration of adoptive UCB-derived NK cells *in vivo* was short, and the number of donor’ NK cells decreased gradually (13, 14), which would limit the role of UCB-derived NK cells. To improve the persistence of UCB-derived NK cells *in vivo*, IL-15 gene transduced into NK cells have been developed, and although these UCB-derived CAR-NK cells were shown to last for 12 months, they were not sufficient to prevent recurrence (3). Thus, it is imperative to develop a strategy that can not only prolong the duration of donor NK cells, but also maintain a relatively high level of donor NK cells *in vivo*. Secondly, the patients received other treatments after infusion of UCB-derived NK cells, therefore, the response duration of infused NK cells could not be assessed, that made it hard to draw a definite conclusion on the clinical efficacy of these UCB-derived NK cells in the treatment of tumor patients (3, 14), therefore, randomized controlled trials should be carried out. Third, the conditioning regimen, the time point of NK cell infusion and the optimal range of NK cell infusion have not been determined, which needs to be further explored. Fourth, studies on the characteristics of allogeneic UCB-derived NK cells in patients who do not have a response or who have a relapse after receiving NK cells therapy should be performed, to find out the potential mechanism of immune escape and provide a novel strategy for engineering NK cells.

## Conclusion

In summary, UCB-derived NK cell-based immunotherapy has great therapeutic potential for cancer patients. Although the number of NK cells is often small and NK cells have immature phenotypes in UCB, the number and function of them are usually significantly improved after expansion *in vitro*. Moreover, expanded UCB-derived NK cells possess high anti-tumor effects both *in vivo* and *in vitro*. Thus, UCB-derived NK cells can be used as an allogeneic “off-the-shelf” product in anti-cancer cellular immunotherapy, making their use a promising option.

## Expert Opinion

UCB-derived NK cells can be viewed as a potential “off-the-shelf” product with the following privileges. UCB is an easily available resource and can be regarded as a third-party platform for the source of NK cells, based on a large number of stored UCB units, including frozen and fresh units (68). Moreover, one study showed that it was possible to produce more than 100 doses of NK cells from a single UCB unit for clinical use, suggesting the UCB-derived NK cells have a strong ability to proliferate *in vitro* (12). Meanwhile, an investigation indicated that long-term cryopreservation did not affect the expansion ability and activity of UCB-derived NK cells, which is another significant advantage (7). Furthermore, in order to fight against tumor cells more effectively and specifically, researchers made attempts to modify genetically UCB-derived NK cells *via* various approaches (12, 40), fortunately, gene-modified NK cells have been successfully developed and can be used to be against tumor cells possessing specific tumor antigens or secreting certain immunosuppressive cytokines (3, 60). Then, they all could be produced in compliance with good manufacturing practice, as an “off-the-shelf” product for clinical use and be used to treat cancer patients. Of note, these UCB-derived NK cells can recognize and kill tumor cells through tumor-specific cell surface receptors and own receptors, which also means that they can kill the on-target/off-tumor cells through their own receptors. Besides, for clinical use, indeed, the existence of the UCB bank ensures that donors of certain HLA types and those with specific NK receptor profiles can be selected.

It’s also worth pointing out that effective adoptive NK cells-based immunotherapy requires NK cells to be activated, sufficient in number, and considerably persistent in the body, to enter tumor sites and effectively to kill tumor cells. More specifically, researchers had developed a variety of UCB-derived CAR-NK cells, which could specifically kill tumor cells according to tumor antigen as CAR-T cells do, showing significant anti-tumor activities. For instance, they have successfully utilized CAR-CD19-NK cells derived from UCB to kill CD19 positive tumor cells (3, 12). Except for it, in particular, based on the mechanism of killing tumor cells mediated by activating and inhibitory receptors of NK cells, UCB-derived NK cells can also be modified to enhance anti-tumor activity by increasing the expression of activating receptors or decreasing the expression of inhibitory receptors, which is a privilege of NK cells. Of note, though, is that while improving the killing effect of NK cells is essential, the possible immunosuppressive effects of tumor microenvironments on the activity of NK cells should also be taken into account. Further, it is also crucial to prolong the duration of NK cells *in vivo* and promote expansion after infusion, therefore, NK cells can be transduced with cytokine-encoding genes, such as the IL-15 gene. Then, to promote homing of NK cells to tumor sites and increase their adhesion to tumor cells, NK cells are modified to express chemokine receptors and membrane-bound scFv directed against tumor-related antigens. In this way, modified UCB-derived NK cells will kill tumor cells more effectively.

Interestingly, from the results of published clinical trials, UCB-derived NK cells could induce responses in AML, MM and high-risk CD19-positive cancers with relatively few adverse events and no GVHD (3, 13, 14). Nevertheless, the issues that remain to be addressed in clinical trials, for instance, how to further improve the duration and number of adoptive UCB-derived NK cells *in vivo* post treatment, and how to accurately evaluate their effectiveness. Moreover, clinical trial design is also a vital determinant of success, such as the time and number of NK cells infusion. Thus, more experimental and clinical investigations are required to develop and optimize UCB-derived NK cell-based anti-cancers therapies demonstrating high efficiency and low toxicities. In the future, based on the fact that UCB-derived NK cells are an “off-the-shelf” product, multimodal therapy consisting of the combination of UCB-derived NK cells with chemotherapeutic drugs or antibodies or HSCT could benefit patients with cancer.

## Author Contributions

HW, YH, and XZ designed this work, and XZ and LC wrote this manuscript. All authors contributed to the article and approved the submitted version.

## Funding

The study was supported by grants from the National Natural Science Foundation of China, No.81770134.

## Conflict of Interest

The authors declare that the research was conducted in the absence of any commercial or financial relationships that could be construed as a potential conflict of interest.
